# Small Area Estimation of Undernutrition under Age Five Children Based on Spatial-Temporal Models

**DOI:** 10.1155/2022/6882047

**Published:** 2022-04-28

**Authors:** Seyifemickael Amare Yilema, Yegnanew A Shiferaw, Temesgen Zewotir, Essey Kebede Muluneh

**Affiliations:** ^1^Department of Statistics, College of Science, Bahir Dar University, P.O Box 79, Bahir Dar, Ethiopia; ^2^Department of Statistics, College of Natural and Computational Science, Debre Tabor University, P.O Box 272, Debre Tabor, Ethiopia; ^3^Department of Statistics, University of Johannesburg, P.O Box 524, Johannesburg 2006, South Africa; ^4^Department of Statistics, School of Mathematics, Statistics and Computer Science, University of KwaZulu-Natal, Durban, South Africa; ^5^School of Public Health, Bahir Dar University, P.O Box 79, Bahir Dar, Ethiopia

## Abstract

The mean flow of direct survey estimates is mainly concerning the sample adequacy fulfillment unless it has been produced large variance estimates, and therefore, the small area estimations are developed to manage this flaw of the path. Small area estimation improved the direct survey estimates by borrowing strength from the census data and at the same time by using historical data from consecutive surveys. In this paper, we applied the spatiotemporal Fay–Herriot (STFH) model for producing fairly reliable disaggregate-level estimates of undernutrition indicators across all zones. The STFH model is an appropriately fitted model to the undernutrition data since it has the lowest information criteria (IC) value. The spatiotemporal estimates improved both the direct and spatial estimates of undernutrition under the FH model and have brought efficiency gain in the percent coefficient of variation (CV). These results may provide useful information to the government's planners, policymakers, and legislative organs for effective policy formulation and budget allocation in all zones.

## 1. Introduction 

The complete enumeration of surveys in geographically small areas with adequate sample sizes is too expensive and time-consuming; it is utterly unthinkable for developing countries like Ethiopia. It is known that censuses are conducted once in a decade, while surveys are conducted within five years of intervals. A survey is often planned to provide reasonable estimates at large geographical areas like national and regional levels [1]. However, the sample sizes are seldom large enough for small areas to produce direct estimates of adequate precision for the domains characteristics of interest [2]. Large estimation errors are produced in such cases, and the inferences are unreliable and useless for policymakers [1, 2].

The demand for small area statistics at disaggregated levels is increasing across the globe for policy interventions [1]. In addition to survey estimates produced efficiently at large scales (national and regional levels), these surveys also contribute to the country's economic, health, social, and political decisions, and policy implementations. The legislative organs of the government of Ethiopia ratified laws, implemented policies, and made political decisions are based on only the information received from the national and regional levels. The estimated information is not decentralized as the government's structures are decentralized. The accessibility of disaggregate-level statistics for target-oriented effective policy planning and monitoring is essential for Ethiopia's decentralized administrative planning systems.

Different researchers have suggested several model-based techniques of small area estimation to improve the direct survey estimates for domains with small samples sizes [3–5]. The ordinary Fay–Herriot (FH) model was developed and studied by [1, 6–9] to improve the small areas with small sample sizes. The spatial small area estimates are studied by [4, 10–13] with area correlations of the characteristics interest under the FH model. The need for spatial information in neighborhood areas is stated by [14] as “everything is related to everything else, but near things are more related than distant things.” Thus, closer areas tend to have similar socioeconomic characteristics of interest than distant small areas. Such extensions of the spatial FH model are further studied by [5, 10, 15].

Spatiotemporal small area estimation incorporates time-related historical data. It simultaneously includes the spatial correlation among data from the neighboring areas (zones in our cases) with fixed regression parameters across time.

In our study, we considered the zones to be small areas. This study focuses on child undernutrition indicators stunting, wasting, and underweight among children under five years of age. Among undernutrition indicators; stunting (height-for-age), underweight (weight-for-age), and wasting (weight-for-height) have been considered. Children whose height-for-age z-score is below minus two standard deviations (−2 SD) from the reference population's median are considered stunted. Stunting is also called shortness, which means low height relative to age. Children whose weight for height z-score is below minus two standard deviations (−2 SD) are considered as wasted [16–18]. This study used the z-scores of the standard forms of stunting, wasting, and underweight as continuous variables to utilize the maximum amount of information available in the data set.

Globally, estimated 144 million and 47 million under five-year-old children were stunted and wasting, respectively, according to a research in [17]. The majority of the world's stunted, underweight, and wasted children under the age of five lived in Asia and Africa [17]. Furthermore, undernutrition is associated with 45 percent of deaths in children under the age of five worldwide [17]. In Ethiopia, 38%, 10%, and 24% of children under the age of five were stunted, wasting, or underweight, respectively [18].

Decentralization is the most important administrative element in Ethiopian healthcare system [19]. Complementary to government institutions, the federal ministry of health decentralized the health service (regions, zones, and woredas). These administrative hierarchies are the most important entities in the country's healthcare delivery [19, 20]. Between regional and woreda (district) governments, zonal governments act as a link (milestone). The health efforts in the districts are monitored and evaluated by the zonal health department [19]. As a result, estimates of undernutrition indicators at the zonal level are a considerable benefit for legislative bodies, policymakers, and monitors at all levels of government.

The focus of this paper is exploiting the spatial information obtained via the neighborhood area characteristics of interest for improving the direct survey estimates for unplanned domains, which are zones. Besides this, the spatiotemporal model has been adopted to further improve the direct survey estimates by simultaneously incorporating the four years from 2000 to 2016 Ethiopian demographic and health survey (DHS) to strengthen the direct survey estimates of the last survey data. The Ethiopian DHS has been carried out within five-year intervals for the large geographical areas of regions and national levels. This study generated a spatiotemporal zonal level estimate using the surveys taken in 2000, 2005, 2011, and 2016. In this study, the researcher applied spatial FH and STFH models to obtain reliable and precise estimates of undernutrition (stunting, wasting, and underweight) by linking characteristics of interest from the 2000, 2005, 2011, and 2016 DHS data, which are considered to be temporal data, with the 2007 census data.

The remainder of the paper has been arranged as follows: in Section 2, the study discussed the methods and materials of the study, the spatial FH model with spatial correlation among the small areas (zones in our case), and the STFH model, which incorporates both area effects and time-related random effects. We report the results of the data analysis in Section 3 and discuss them in Section 4. Finally, we present conclusions in Section 5.

### 1.1. Literature Review

The spatiotemporal model in small area estimations is proposed by [5] and further studied by [21–23]. Using survey data from 2004 to 2008, a spatial-temporal Fay–Herriot model is applied with Spanish data to estimate poverty indicators for Spanish provinces in 2008 [5]. The spatial autocorrelation among the neighboring areas might be exploited to improve the direct survey estimates; however, incorporating time-related historical data further improves the direct survey estimates and spatial small area estimates [5, 21]. The spatiotemporal small area estimation is not studied in the country, yet it has not received attention in the undernutrition literature.

Estimation results for spatial and spatiotemporal small area models were compared in different types of literature [5, 21, 23, 24]. The spatiotemporal small area estimates of income in Poland data were applied by [25]. According to the studies in [25], the spatiotemporal models that used spatial correlation between neighboring areas as well as historical data were compared to EBLUPs based on spatial models derived separately for each year and with EBLUPs [5, 22, 23, 26]. The findings, the Polish data coming from the household budget survey and the administrative data, show that spatiotemporal small area models has been realized a noticeable reduction in estimation errors, especially when strong spatial and time autocorrelations were detected [25].

Spatiotemporal Fay–Herriot models are one of the SAE approaches that have incorporated spatial and time effects and have been utilized in poverty at the district level in west Sulawesi province [22]. The studies in [27] investigated area-level time models for small area estimate of poverty indicators and borrowed strength from time by employing area-level linear time models. The Spanish living conditions survey's poverty indicators are evaluated using spatiotemporal models [27]. Spatiotemporal Fay–Herriot model applied with Spanish EU-SILC data is carried out to obtain estimates of poverty indicators for Spanish provinces in 2008, making use of survey data from years 2004–2008 [21]. Spatiotemporal data contain a diverse set of variables, posing distinct problems and possibilities for professionals attempting to maximize its full potential.

## 2. Methods and Materials

### 2.1. Data Sources

The data were taken from the nationally representative cross-sectional study design of 2000, 2005, 2011, and 2016 Ethiopian DHS for the characteristics of interest and the 2007 census data for auxiliary variables. The Ethiopian DHSs are designed nationally representative, probabilistic, and household surveys that include a wide range of key demographic and health indicators used to monitor and evaluate population, health, and nutrition programs [18, 28–30]. The 1994 and 2007 population and housing census were considered for the sampling frame classifications, and the collection of the surveys is based on standardized questionnaires that yield different data files [18, 28–30]. Within 83 zones in Ethiopia, this study was conducted on under five-year-old children consisting of 8590 under five-year-old children from 2000, 3874 under five-year-old children from 2005, 9611 under five-year-old children from 2011, and (8505 stunting, 8675 wasting, and 8556 underweight) from 2016 Ethiopian DHS data. The height and weight measurements were collected from children 0–59 months [18, 28–30] in all the selected households for all survey years.

### 2.2. Study Variables

For this analysis, there are 41 area (zonal)-level proportions of covariates taken from the 2007 population and housing census. Stepwise regression analysis was used for all variables to filter out some of the best explanatory variables. Women aged 15–24, children aged 4–5, parents without disabilities, marital status (separated, widowed, divorced, and others), illiterate mothers, mothers with babies younger than one, and families with only one daughter who has died are selected for stunting under five-year-old children. Females, children under one year old, different marital statuses (separated, widowed, divorced, etc.), parents without disabilities, children ages 2–3, and mothers working for governmental organizations are targeted for wasting under age five children. Families with less than age five children, other marital statuses (separate, widowed, divorced, etc.), married, improved water facilities, and other occupations are selected for underweight children under five years of age.

### 2.3. Spatial Data

For administration purposes, Ethiopia has been divided into nine regions and two administrative cities, which, in turn, are divided into 83 zones. The global positioning system (GPS) point data were linked to each sampled urban-rural cluster residence to all household attributes. The GPS urban/rural locations have been masked [31] for confidentiality reasons. The GPS latitude/longitude position for DHSs data is randomly displaced to keep the respondent's confidentiality. In small area administrative units, the displacement is randomly carried out with 2 kilometers, and 5 kilometers for urban and rural residence clusters, respectively, and also 1% of rural clusters were displaced up to 10 kilometers [31]. The GPS point data as shapefiles are also available and obtained from https://www.dhsprogram.com. And also, the shapefiles for Ethiopian administrative boundaries are available on the website https://africaopendata.org.

### 2.4. Spatial and Spatiotemporal Small Area Estimation

The FH model has been extended in various works of literature. The multivariate FH models are investigated by multiple researchers [3, 6, 32–36]. The temporal FH model, which borrows strength from historical data, past time instants, and correlations, is studied by [27, 37] to produce reliable area-level estimates. The STFH model borrowed strength from census data and similar small areas through the time effect in historical data and spatial effects [5, 21, 23]. In the STFH model, the spatial and time-related dependencies have had between domains (zones in this case).

Let *θ*_*it*_ be the variable of characteristics of interest for area *i* and time *t*, where *i*,…, *m* and *t*,…, *T*. If the direct estimator of this quantity is denoted by ${\hat{\theta }}_{it}^{\text{dir}}$, the sampling errors can be expressed as *ε*_*it*_. Similar with the FH model, the extended STFH model has two stages. The first stage can be expressed as follows:

(1) $$\begin{array}{c} {\hat{\theta }}_{it}^{\text{dir}}=\;\theta_{it}+\varepsilon_{it}, \end{array}$$

where the sampling error *ε*_*it*_ is assumed to be independent and normally distributed with variance *σ*_it_^2^ known for all *i* and *t*.

The small area model, which incorporates the spatiotemporal relationships in the second stage, is given as

(2) $$\begin{array}{c} \theta_{it}=\;X_{it}^{T}+\nu_{1i}+\nu_{2i}, \end{array}$$

where *X*_*it*_ is the vector of *p* auxiliary variables dependent linearly with *θ*_**it**_ for area *i* at time instant *t*, and *β* is the regression coefficients of auxiliary variables. Finally, the area effects vector (*ν*_11_,…,*ν*_1*m*_)^*T*^ follows a first-order simultaneous autoregressive process with variance parameter  *σ*_1_^2^, and spatial autocorrelation |*ρ*_1_| ≤ 1 and row-standardized proximity matrix *W*. And also the vectors of area-time random effects (*ν*_21_,…,*ν*_2*m*_)^*T*^ will follow identically and independently distributed for each area *i* and follow an first order autoregressive (AR(1)) with autocorrelation parameter |*ρ*_2_| ≤ 1, that is,

(3) $$\begin{array}{c} \nu_{2i}=\;\rho_{2}\;\nu_{2i,\;t-1}+\varepsilon_{2it}, \end{array}$$

where *ε*_2*it*_ ~ *ii*  *d* *N*(0, *σ*_2_^2^).

The matrix notations of STFH general linear mixed model can be written as

(4) $$\begin{array}{c} y=X\beta +Z\nu +\varepsilon . \end{array}$$

Using stacking notations for vectors and matrices, the following relationship is considered as $y=\;{\text{col}}_{1<i<m}\left({\text{col}}_{1<t<T}\left({\hat{\theta }}_{\text{it}}\right)\right)$, **X**=col_1<*i*<*m*_(col_1<*t*<*T*_(*X*_it_)), *ε*=col_1<*i*<*m*_(col_1<*t*<*T*_(*ε*_it_)), *ν*_1_=col_1<*i*<*m*_(col_1<*t*<*T*_(*ν*_1*i*_)), and *ν*_2_=col_1<*i*<*m*_(col_1<*t*<*T*_(*ν*_2*i*_)), where *ν*=(*ν*_1_^*T*^, *ν*_2_^*T*^)^*T*^, and **Z** is the constant unit matrix. Let the random component parameter *τ*=(*σ*_1_^2^, *ρ*_1_, *σ*_2_^2^ , *ρ*_2_)^**T**^ be the vector of unknown parameters involved in the covariance structure of the STFH model. The random sampling errors *ε* ~ *N*(0, *V*_*ε*_), where 0 vectors of zeros, and *V*_*ε*_ is the diagonal matrix *V*_*ε*_=diag_1<*t*<*T*_(*σ*_it_^2^). In addition, *ν* ~ *N*(0, **V**_*ν*_(*τ*)) with covariance matrix given by the block diagonal matrix has the following form **V**_*ν*_(*τ*)=diag(*σ*_1_^2^Ω_1_(*ρ*_1_),  *σ*_2_^2^Ω_2_(*ρ*_2_)), the matrices Ω_1_ and Ω_2_ have the following relationships Ω_1_=[(*I*_*m*_ − *ρ*_1_*W*)^*T*^(*I*_*m*_ − *ρ*_1_*W*)]^−1^ and Ω_2_=diag_1<*i*<*m*_Ω_2*i*_(*ρ*_2_), and

(5) $$\begin{array}{c} \Omega_{2i}\left(\rho_{2}\right)=\;\frac{1}{1-\rho_{2}^{2}}\left[\begin{array}{ccccc} 1 & \rho_{2} & \ldots & \rho_{2}^{T-2} & \rho_{2}^{T-1}\\ \rho_{2} & 1 & \ldots & \rho_{2}^{T-3} & \rho_{2}^{T-2}\\ \vdots & \vdots & \ddots & \vdots & \vdots \\ \rho_{2}^{T-1} & \rho_{2}^{T-2} & \ldots & \rho_{2} & 1 \end{array}\right]. \end{array}$$

Give all the above expressions, the covariance matrix for the full model (the sampling error plus the random components) can be written as var(**y**)=**Z***V*_*ν*_**Z**^*T*^+*V*_*ε*_.

Note that the STFH model is a more general model and the ordinary spatial FH model can be obtained by ignoring the random time effects. When the spatial autocorrelation *ρ*_1_ and area-time random effects *ρ*_2_ are zero, the STFH model absolutely becomes the complementary FH model [21, 26].

#### 2.4.1. Parameters Estimation of Spatiotemporal Model

Predicting and measuring the variability of the random components is one of the main issues in small area estimations. In this paper, we dealt with the problem of predicting *θ*_it_ by using empirical best linear unbiased prediction (EBLUP) of ${\hat{\theta }}_{\text{it}}$. By adopting the STFH model analogous to the Prasad and Rao [8,38], the mean square error (MSE) of EBLUP estimator under spatiotemporal FH model is as follows:

(6) $$\begin{array}{c} MSE\left({\hat{\theta }}_{it}\right)=g_{1}\left(\hat{\tau \;}\right)+g_{2}\left(\hat{\tau \;}\right)+2g_{3}\left(\hat{\tau \;}\right), \end{array}$$

where $\hat{\tau }={\left({\hat{\sigma }}_{1}^{2},{\hat{\rho }}_{1},{\hat{\sigma }}_{2}^{2}\;,{\hat{\rho }}_{2}\right)}^{T}$ is the vector of estimated random variance components in the STFH model, $g_{1}\left(\hat{\tau \;}\right)$ is due to the estimation of random area effects with the order of *O*(1) for large *m*, $g_{2}\left(\hat{\tau \;}\right)$ is due to the estimates of *β* with order *O*(*m*^−1^), and the third term $g_{3}\left(\hat{\tau }\right)$ is due to the estimates of variance component.

The STFH model is fitted by restricted maximum likelihood (REML) methods, and also, the parametric bootstrap techniques are used for estimating the spatiotemporal EBLUP and its MSE [5, 21, 26]. The R package sae provides small area estimation methods based on the area-level models extended Fay–Herriot model, which allows for spatiotemporal correlation [39].

## 3. Result

### 3.1. Diagnostic Measures

The random component parameter estimator of the STFH model is reported in Table 1. The spatial correlations under the simultaneous autoregressive process are 96.5, 90.5, and 98 for stunting, wasting, and underweight, respectively. In addition, the time-related autoregressive are −63, −68.7, and −73.3 for stunting, wasting, and underweight, respectively. These results show that both the spatial area effect and the time-related random effects are in the FH model, and therefore, STFH model is appropriate model of this analysis. The random component variance of both the spatial and times effects is also presented in Table 1 for all target variables.

The relative best model was identified using the information criterion (IC) in Table 2. The IC (−2 log-likelihood (−2LL), Akaike information criterion (AIC), and Bayesian information criterion (BIC)) of the STFH model are smaller than the spatial Fay–Herriot model (SFH) for all undernutrition indicators. Thus, the STFH model is better than the SFH model.

The *p*-values for stunting, wasting, and underweight are a lot larger than 0.05 for the Kolmogorov–Smirnov test. Thus, we can conclude that the distribution of the STFH model for the undernutrition indicator does not differ significantly from a normal distribution. As a result, the STFH model meets its normality assumption satisfactorily.

**Figure 1:**
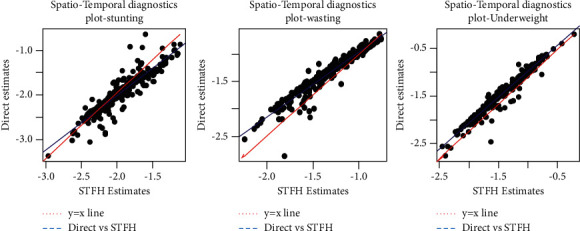
Bias diagnostic plot with *y* = *x* line (red line) and regression line (blue line) for stunting, wasting, and underweight: Model-based STFH estimates versus direct estimates.

Figure 1 shows the STFH model bias diagnostics measures. The direct survey estimates are plotted on the *y*-axis, and the spatiotemporal estimates are plotted on the *x*-axis. The diagnostic measure examines the validities of model-based spatiotemporal small area estimates. The regression of direct survey estimates is analogous to the spatiotemporal small area estimates since the spatiotemporal estimates are adjacent to the actual values. The graph shows that the model-based spatiotemporal small area estimates are not very far off from the fitted values of the regression line. As a result, the spatiotemporal estimates of small areas are not very different from direct survey estimates, indicating that the model-based estimates are valid. Overall, the bias diagnostic measures indicate that the model-based spatiotemporal small area estimates are likely to agree with direct survey estimates for all target variables of undernutrition indicators less than five years.

**Figure 2:**
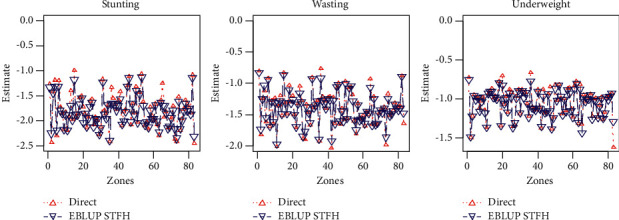
EBLUPs based on STFH model and direct estimates of children under five years of age for each zones.

Figure 2 shows the STFH-based EBLUP estimates and the direct estimates. According to the figure, EBLUPs and direct survey estimates are equivalent, so EBLUPs are stable in all undernutrition indicators (stunting, wasting, and underweight).

**Figure 3:**
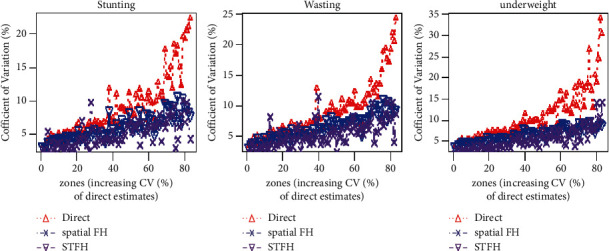
Zones percentage of coefficient of variation (CV) of direct, spatial FH, and STFH estimators of undernutrition indicators.

The percent coefficient of variation of spatiotemporal small area estimates, spatial small area estimates, and direct estimates are presented in Figure 3. The percent coefficient of variation (CV) of spatiotemporal EBLUP is smaller than the corresponding direct estimates and spatial EBLUP estimates. From the figure, we observed that the CV (%) of direct survey estimates of undernutrition indicators is larger than the corresponding spatial small area estimates. The spatial small area estimates are more precise and reliable than the direct survey estimates, meaning that spatial estimates improve the direct survey estimates because of the correlation among the neighboring zones.

Compared with the spatial estimates, the spatiotemporal estimates are deemed more precise and reliable. The spatiotemporal small area estimates improve the precision of spatial estimates for all undernutrition indicators. In addition to being more precise than direct survey estimates, spatiotemporal small area estimates also improve them. As a result, the direct survey estimates and spatial small area estimates are improved by spatiotemporal small area estimates. Generally, using temporal data (2000, 2005, 2011, and 2016 Ethiopian DHS) with fixed regression parameters from census data across all surveys improves the direct estimates and spatial small area estimates. Due to the inclusion of time-related autoregressive AR (1) correlations across small areas, STFH models have an advantage over spatial FH models.

**Figure 4:**
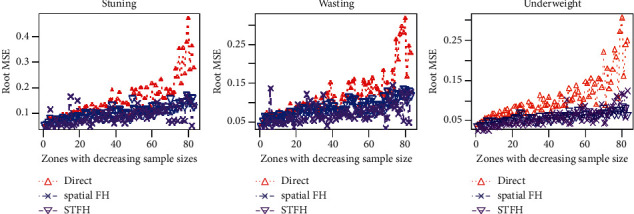
Zones (increasing sample size) Root MSE of direct and STFH estimators of stunting, wasting, and underweight.

Figure 4 show the zonal-wise root MSE of stunting, wasting, and underweight for children under five years of age. Direct survey estimates have the largest root MSE, while spatiotemporal EBLUPs have the smallest root MSE. Because the root MSE means are small, spatiotemporal estimates are the most precise, followed by spatial small area estimates. By contrast, direct survey estimates are the least accurate. Consequently, we can confirm that the spatiotemporal EBLUP is the most reliable and precise estimate of undermatron indicators in children under age five.

The summary results of spatiotemporal EBLUP efficiency gain in CV over the spatial EBLUP and direct survey estimates are presented in Table 3. This table examined the magnitude to which the spatiotemporal small area estimates of stunting, wasting, and underweight improved in precision than the spatial EBLUP and direct survey estimates. The efficiency gains in CV due to spatiotemporal EBLUP over the direct and spatial EBLUP are improved for all target variables. Compared to direct survey estimates, the spatiotemporal EBLUP methods showed a median value of 53.74% with a maximum value of 90.34% for stunting, a median value of 49.60% with a maximum value of 91.30% for wasting, and a median value of 49.23% with a maximum value of 89.64% for underweight. The minimum, the first quartile, the mean, and the third quartiles of spatiotemporal EBLUP efficiency gain in CV over direct survey estimate for all undernutrition indicators are reported in Table 3. A few zones have a loss in efficiency for all target variables since the minimum values of gain in efficiency are recorded as negative values.

The spatiotemporal small area estimates efficiency gain over the spatial small area estimates are also investigated and reported in Table 3. The results of spatiotemporal EBLUP have a maximum value of 78.41%, 77.76%, and 68.77% efficiency gain for stunting, wasting, and underweight, respectively, over the corresponding spatial EBLUP. These results clearly show that the spatiotemporal small area estimates are more precise, efficient, and reliable than corresponding to the spatial small area for stunting, wasting, and underweight due to the incorporations of temporal effects on the spatially correlated zones. The spatiotemporal small area estimates improved the direct survey estimates and the spatially correlated zonal estimates. Therefore, the spatiotemporal small area estimates are the best reliable, precise, and efficient estimates for all undernutrition indicators.

## 4. Discussion

This part discussed the spatiotemporal small area estimates of undernutrition indicators: stunting, wasting, and underweight for children under age five. This article provides the zonal estimates of undernutrition indicators: stunting, wasting, and underweight for children under age five in Ethiopia using four consecutive surveys (2000, 2005, 2011, and 2016) and the 2007 population and housing census data. The standardized z-scores of undernutrition indicators, stunting, wasting, and underweight were used to exploit the maximum amount of information. The STFH model was applied to obtain zonal level estimates of undernutrition indicators in Ethiopia. It is a STFH model that accounts for the spatial correlation between neighboring areas and that simultaneously incorporates the time-related (four consecutive surveys from *T* time instants) to enhance small area estimates at the current time (in this case, the 2016 survey) [5, 21, 23].

The validity, reliability, and precision of model-based spatiotemporal small area estimates of undernutrition indicators were examined using bias diagnostics, Kolmogorov–Smirnov test, CVs, and root MSEs [2, 5, 21–23]. These measures indicate that the spatiotemporal small area estimates are superior to the direct survey estimates since the STFH model borrows strength from time-related temporal data from the four consecutive surveys (2000, 2005, 2011, and 2016) [5, 21–23]. In addition, the Kolmogorov–Smirnov test and the bias diagnostics of spatiotemporal small area estimates versus direct survey estimates (Figure 1) were used to test the model assumptions. Therefore, the model assumptions have been met satisfactorily.

For comparison, we computed the percentage CVs and root MSEs of direct survey estimates, spatial small area estimates, and spatiotemporal small area estimates of undernutrition indicators. In comparing the percentage CV of direct estimates and model-based spatiotemporal estimates (Figure 3), the spatiotemporal estimates were lower than the direct estimates for all indicators of undernutrition. Besides comparing spatiotemporal small area estimates with direct survey estimates, we also compared the percentage CV of the spatiotemporal estimates with the spatially correlated zonal estimates (Figure 3). Consequently, the spatiotemporal estimates have a lower CV than the spatial small area estimates. Similarly, the comparisons of root MSEs follow a CV-like approach.

As a result of the model assumptions and small area estimate diagnostic, the spatiotemporal small area estimates were more stable and precise than the corresponding direct survey estimates and spatial small area estimates for all undernutrition indicators of children under age five. And in turn, the spatial small area estimates have greater precision and reliability than the direct survey estimates [4, 12, 40]. Direct survey estimates of undernutrition have improved using a spatial FH model with reasonably large spatial autocorrelations. And also, the spatiotemporal model further enhances the direct survey estimates, taking into account the temporal data considerations.

As a measure of the performance estimators under the most general model, the STFH model, efficiency gains in the CV of the STFH model over the spatial FH model, and direct estimates are computed. The average median improvements in the CV of the STFH model over the direct survey estimates are 53.74, 49.60, and 49.23 for stunting, wasting, and underweight, respectively (Table 3). Similarly, the average median efficiency gains in the CV of the STFH model over the spatial FH model are 33.78, 28.07, and 15.66 for stunting, wasting, and underweight, respectively (Table 3). Thus, it is evident that using temporal data in the STFH model offers advantages over spatial estimates of undernutrition indicators such as stunting, wasting, and underweight [5, 21].

## 5. Conclusion

This paper applied the STFH methods in four consecutive surveys and Ethiopia's 2007 population and housing census data to improve the direct survey estimates of undernutrition indicators for children under age five across all zones. The CSA conducted regular surveys on several essential health, demographic, and socioeconomic indicators, but the results were limited to national and regional estimates. By contrast, the administrative levels below regional levels are not examined due to small sample sizes. As this paper demonstrated, spatiotemporal small area estimations can be used as a cost-effective and efficient method for estimating undernutrition indicators.

The improvement of direct survey estimates and spatial small area estimates of zones are achieved in root MSE and CV for all target variables. It is evident that the use of temporal data in the ST FH model brings efficiency gain in CV over the spatial small area estimates of undernutrition indicators stunting, wasting, and underweight. Therefore, the use of temporal data is adequate. Consecutively, STFH models have an advantage over spatial FH models with temporal consideration due to the inclusion of time-related correlations across the zones. These results may provide useful information to the government's planners, policymakers, and legislative organs for effective policy formulation and budget allocation in all zones.

## Figures and Tables

**Table 1:** Parameters estimator of STFH model.

Parameters	Stunting	Wasting	Underweight
*σ*_1_^2^	0.00019	0.0026	0.0014
*σ*_2_^2^	0.0106	0.044	0.08
*ρ*_1_	0.965	0.905	0.98
*ρ*_2_	−0.63	−0.687	−0.733

**Table 2:** Model comparison based on information criteria.

IC	Spatial FH model			STFH model		
	Stunting	Wasting	Underweight	Stunting	Wasting	Underweight
−2LL	384.52	240.72	367.1	171.56	135.7	288.26
AIC	210.27	262.72	389.09	195.57	159.69	312.24
BIC	248.32	304.57	430.95	241.23	205.35	357.90

**Table 3:** Summary statistics of efficiency gain in CV (%) for spatiotemporal EBLUPs over direct survey estimates and spatial EBLUPs.

	Efficiency gains of STF model over direct estimates			Efficiency gains of STFH model over the spatial FH model		
	Stunting	Wasting	Underweight	Stunting	Wasting	Underweight
Min	−132.84	−118.31	−117.60	−139.28	−145.30	−181.122
Q1	34.03	35.42	29.65	11.75	8.01	−10.44
Mean	47.61	43.96	39.39	25.68	20.39	4.79
Median	53.74	49.6	49.23	33.78	28.07	15.66
Q3	71.09	66.64	64.11	47.36	44.73	37.16
Max	90.34	91.30	89.64	78.41	77.76	68.77

## Data Availability

The 2000–2016 Ethiopian DHS data were accessed after a request for registration from the DHS program website https://www.dhsprogram.com. Similarly, the GPS enumeration area shape files were obtained from https://www.dhsprogram.com, and also the shape files for Ethiopian zonal-level administrative boundaries are available on the website https://africaopendata.org. The Central Statistical Agency in Ethiopia provides access to 10 percent sample of the 2007 census for research purposes.

## Abbreviations

AIC: Akaike information criteria
BIC: Bayesian information criteria
CV: Coefficient of variation
DHS: Demographic and health survey
EBLUP: Empirical best linear unbiased predictor
FH: Fay–Herriot
GPS: Global positioning system
IC: Information criteria
LL: Log likelihood
MSE: Mean square error
REML: Restricted maximum likelihood
SD: Standard deviation
STFH: Spatiotemporal Fay–Herriot.
